# Long Noncoding RNA in Preeclampsia: Transcriptional Noise or Innovative Indicators?

**DOI:** 10.1155/2019/5437621

**Published:** 2019-04-14

**Authors:** Xiuhua Yang, Tao Meng

**Affiliations:** Department of Obstetrics, The First Hospital of China Medical University, Shenyang, Liaoning, China

## Abstract

Preeclampsia (PE) is termed as an obstetric issue that is characterized by hypertension (≧140/90 mm Hg), together with proteinuria following 20 weeks of pregnancy. Until today, PE still constitutes a severe threat to the lives of both the mothers and fetuses. In the past, long noncoding RNAs (lncRNAs) were considered as the transcriptional noise. However, some investigations have indicated that lncRNAs could be used as innovative indicators in PE. The current review aims to discuss the relationship between lncRNAs and PE in recent years. According to the retrieved data, we concluded that lncRNAs can exert an impact on both the occurrence and development of PE through the changes in the biological functions of trophoblasts, immune regulation, epigenetic regulation, decidualization, and energy metabolism. The mechanisms of lncRNAs in PE will help us to better understand the pathogenesis of PE and help us to find targets for predicting and diagnosing PE in the future.

## 1. Introduction

Preeclampsia (PE) is termed as an obstetric issue that is characterized by hypertension (≧140/90 mm Hg), together with proteinuria following 20 weeks of pregnancy [1]. The incidence of PE is 5-8%, and it may involve the liver, kidney, cardiovascular system, cerebrovascular system, and blood system [2]. Until today, PE still constitutes a severe threat to the lives of both the mothers and fetuses. Females with PE are prone to an augmented threat of developing cardiovascular illnesses in the future [3]. Besides that, it is held that the underlying causes of PE count on the inflammatory elements, in addition to the damaged endothelial function, vasoactive elements, oxidative stress, and genetic causes [4–8]. Nowadays, the “Two-phase Disorder” [9] statement of PE is quite famous. In the preliminary phase of the embryonic implantation, trophoblasts invade the uterine wall, besides helping with uterine artery remodeling. There is an increase in the diameter of blood vessels, together with the decline in the blood flow resistance, aimed at ensuring a sufficient amount of blood flow [10]. Trophoblast cells are quite pivotal for uterine spiral artery remodeling. In the patients, who have PE, the invasive potential of trophoblasts declines, causing the dysplasia of uterine spiral artery remodeling, further leading to a shallow placental implantation [11]. Nowadays, the effective treatment for PE is still regarded as the termination of pregnancy; nevertheless, it does not constitute a reasonable option in case of too few numbers of gestational weeks, coupled with an immature fetus [12]. There have been carried out several investigations primarily emphasizing the physical and biochemical indicators for the forecast, diagnosis, and management [13]. Nevertheless, no exclusive indicator could be put to use as a standard and productive indicator. Accordingly, it is quite essential to investigate the pathogenesis for the purpose of finding a means of preventing and treating PE [14].

Long noncoding RNAs (lncRNAs) regulate the gene expression at the transcriptional level and the posttranscriptional level [15]. In the past, lncRNAs were considered as the transcriptional noise. At present, there are several evidence showing that lncRNAs are associated with both the occurrence and development of numbers of illnesses, for instance, cancer [16], cardiovascular illnesses [17], and neurological illnesses [18]. Besides that, a few investigations have suggested that lncRNAs take part in numerous cell biological procedures that include cell proliferation, together with cell migration, cell apoptosis, X-chromosome inactivation, gene imprinting, and stem cell transformation [19–22]. LncRNAs contribute by means of a number of mechanisms. They function as scaffolds, signals, and antisense decoys, in addition to participating in the transcriptional disturbance. Single lncRNA less often performs multiple functions [23]. Scaffold lncRNAs bring together proteins and other RNAs for becoming a bigger functional compound; for instance, the telomerase RNA compound was required for the telomere repeat complex [24], and the polycomb repressor complex was needed for the histone regulation [25]. The transcription of a few lncRNAs could manifest the silencing of colocated protein-coding genes [26] or function as an indicator of the upstream transcriptional element. Decoy lncRNAs could function through the combing of a target protein, impeding the protein from functioning as it requires [27] or through combing and separating of small regulatory RNAs, for instance, microRNAs (miRNAs) [28]. In comparison with messenger RNAs (mRNAs), the superiority of targeting lncRNAs indicates that it is capable of causing changes in several downstream signal pathways, accordingly carrying out various functions [29]. The demerits associated with being capable of playing a role in multiple downstream pathways are that it may give rise to several unintended damaging effects. That is why, the role of lncRNAs requires carefully understanding before they could be put to use as a therapeutic indicator. In comparison with a normal pregnancy, there were a number of differentially expressed lncRNAs in the placentas of PE patients, which suggested that these lncRNAs might be associated with PE [30]. Besides that, several investigations have demonstrated that lncRNAs could take part in the progress of PE by impacting on the function of trophoblasts. Accordingly, the current review aims to discuss the relationship between lncRNAs and PE in recent years, besides putting efforts for the discovery of the pathogenesis of PE from a new perspective.

## 2. LncRNA Microarray Study in PE

Nowadays, people have discovered many lncRNAs when compared with protein-coding genes in the genome [31]. Owing to the development of the next-generation sequencing technology, increasing numbers of lncRNAs have been discovered. There was an age-matched lncRNA microarray study. The empirical group comprised 6 patients having the early-onset PE (onset before 34 weeks) and the control group was constituted by 6 patients with a premature delivery. The investigation discovered that there were 15,646 upregulated and 13,178 downregulated lncRNAs in the placenta tissues of early-onset PE patients [30]. For the purpose of exploring the underlying functions of these differentially expressed lncRNAs, GO analysis was performed, which subsequently revealed that the pathways associated with cell migration were markedly enriched [30]. Another microarray investigation highlighted 738 differently expressed lncRNAs in the placentas of PE in comparison with normal pregnancies [32]. LOC391533, LOC284100, and CEACAMP8 were confirmed to be increased in the placentas of PE, besides being discovered linked to both the lipid metabolism and angiogenesis [32]. Moreover, it was also discovered that there were 163 differentially expressed lncRNAs in late-onset PE (onset over 34 weeks) placentas in comparison with the normal placentas [33]. NONHSAT116812 and NONHSAT145880 both might be utilized as indicators for PE since they were also confirmed in plasma specimens [33].

## 3. LncRNA RNA Sequencing (RNA-Seq) Study in PE

RNA-Seq is a better method than microarray to discover new lncRNAs [34, 35]. Liu S. et al. found some lncRNAs associated with PE, together with discovering that the jak-stat pathway was related to the etiology of PE [36]. Jing Tong et al. performed another RNA-seq study who collected decidual tissues from normal pregnancy (n=3), early-onset PE patients (n=3), and late-onset PE females (n=3), and the results showed that there were 32 aberrant lncRNAs between early-onset PE and normal pregnancy, 53 differentially expressed lncRNAs between late-onset PE and normal pregnancy, and 32 differentially expressed lncRNAs between early-onset PE and late-onset PE, demonstrating that the pathogenesis of early-onset PE was different from that of late-onset PE [37].

## 4. LncRNAs Affect Cellular Functions of Trophoblast Cells

The deficiency of uterine spiral artery remodeling is the early cause of PE. The capabilities of trophoblast migration and invasion have a close association with epithelial-mesenchymal transition (EMT), which is related to the placental development [38]. The decreased trophoblast proliferation, migration, invasion, and stimulated apoptosis constitute the pivotal reasons leading to PE [39]. LncRNAs are capable of impacting both the occurrence and development of PE through the modification of these functions of trophoblast cells. Some lncRNAs were discovered as increasing in PE (Table 1). The increased RPAIN inhibited the trophoblast invasion, together with inducing the apoptosis through regulating C1q, accordingly promoting the aggravation of PE [40]. Moreover, increased lncRNA uc.187 in the placentas of PE patients promoted the progression of PE not just through the reduction of cell proliferation and invasion but also induced cell apoptosis [41]. The upregulation of lncRNA CCAT1 was carried out in PE, which had the ability to induce the progression of PE by decreasing the level of CDK4 [42]. There was an increase in the expression of lncRNA PRNCR1 in PE as well [43]. As discovered, the mechanism of lncRNA PRNCR1 involved the regulation of the Mitogen-activated protein kinase (MAPK) signaling pathway [43]. There was observed an increase in lncRNA DLX6-AS1 in the placentas of PE patients, leading to PE through the manipulation of the miR-376c/GADD45A expression [44]. The SPRY4-IT1 upregulation substantially decreased cell migration and proliferation, meanwhile still increasing cell apoptosis [45]. LncRNA STOX2-IT3 might decrease the differentiation and invasion of trophoblasts through the regulation of STOX2, followed by making a contribution towards PE [46]. LINC-HELLP was associated with the familial HELLP in Dutch [47]. LINC-HELLP was discovered to be linked to trophoblast proliferation and invasion, and LINC-HELLP mutation could attenuate the differentiation of trophoblasts [48]. LncRNA uc003fir was upexpressed in the vessels of PE placentas in comparison with the normal ones through the regulation of “HIF1-*α*–lncRNA uc003fir–CCL5” axis, which resulted into the development of PE [49].

In contrast, the downregulation of some lncRNAs was also confirmed in PE (Table 2). LncRNA MALAT-1 was substantially reduced in the placentas of PE patients, inducing cell proliferation, meanwhile decreasing the cell cycle and cell apoptosis [50]. In addition to that, lncRNA‐ATB was manifested to be associated with the incidence of PE by means of the manipulation of the procedure of the cell invasion, coupled with the development of the endothelial vessels [51]. The decline in small nucleolar RNA host gene 5 (SNHG5) in the placentas of PE women indicated that SNHG5 induced not only cell proliferation, but also cell invasion, and migration through the manipulation of the miR‐26a‐5p/N‐cadherin axis [52]. The expression of lncRNA maternally expressed gene 3 (MEG3) in PE placentas was decreased as well [53]. The downregulation of lncRNA MEG3 had the potential of inhibiting cell migration, inducing cell apoptosis, and increasing the expressions of NF-*κ*B, Caspase 3, and Bax, further causing the dysplasia of uterine spiral artery [53]. LncRNA TUG1 was decreased in the placental tissues of PE females in comparison with the levels in normal ones. Moreover, the downregulation of TUG1 resulted into the inhibitory functions, which attenuated cell proliferation, migration, and invasion together with increasing the apoptosis in trophoblast cells, and further having a role in spiral artery remodeling by means of increasing Ezh2 and decreasing RND3 level in PE [54]. LncRNA RP11-465L10.10 was decreased in the placentas of females in early-onset PE by targeting MMP9 [30]. In addition, functional investigations revealed that HOXA11-AS was capable of increasing Ezh2 and lysine-specific demethylase 1 (LSD1) and regulating RND3 level in the nucleus, while in the cytoplasm, HOXA11-AS regulated the HOXA7 level via sponged miR-15b-5p, which changed cell proliferation [55]. H19 was decreased in the early-onset severe PE through the regulation of miR-675/Nodal Modulator 1 (NOMO) axis and Nodal signal pathway, and H19 was capable of increasing cell proliferation [56]. The decreased level of EGFR Antisense RNA1 (EGFR-AS1) in PE had the potential to decrease the level of EGFR in trophoblast cells and the phosphorylation levels of downstream proteins in JAK/STAT signal pathway, followed by inducing the occurrence of PE [57]. The level of linc00473 was decreased in the placental tissues of severe PE women, linc00473 downregulation in trophoblast cells substantially reduced cell proliferation, together with increasing cell apoptosis, while linc00473 upregulation increased cell proliferation through the attenuation of tissue factor pathway inhibitor 2 (TFPI2) by combing to LSD1 [58]. LncRNA PVT1 downregulation substantially decreased cell proliferation; besides inducing the cell cycle as well as cell apoptosis, the mechanism of PVT1 was through the regulation of angiopoietin-like 4 (ANGPTL4) as well as Ezh2 [59]. The downregulation of MVIH in PE decreased not only cell proliferation, but also cell migration, invasion and angiogenesis in trophoblast cell lines by targeting Jun-B protein [60].

## 5. LncRNAs Affect Immune Response in PE

The expression level of dendritic cells (DCs) for the production of T regulatory (Treg) cells in PE patients substantially declined [61]. Lnc-DC just appeared in DCs, and functional experiments have indicated that the downregulation of lnc-DC damaged the differentiation of monocytes into DCs, accordingly performing the attenuation of the production of Treg cells by DCs. Lnc-DC could increase the phosphorylation of tyrosine-705 in the cytoplasm, followed by impacting the transcription of downstream genes and stimulating the differentiation as well as the development of DCs [62]. In addition, lnc-DCs stimulated the development of decidual DCs in PE females, besides contributing to a rise in Th1 cells, leading to the etiology of PE [63]. MALAT1-upregulated mesenchymal stem cells (MSCs) induced M2 macrophage polarization and the function was regulated by MALAT1-produced IDO level, which suggested that MALAT1 might induce the immunosuppressive potential of MSCs in vivo [64].

## 6. LncRNAs Affect PE by Epigenetic Regulation

LncRNAs had the potential of mediating the gene expression at the epigenetic level [65]. Moreover, the alternative epigenetic mediation of the H19-IGF2 domain in placental tissues was associated with PE, resulting into placental dysplasia in the early pregnancy [66]. LncRNA H19 rs217727 polymorphism was related to a higher chance of having PE [67]. Moreover, not only the CTC, but also TTC and TTT haplotypes had an association with the PE susceptibility [67]. STOX2-IT3-lncRA performs the role of a permissive cis-acting regulatory factor of STOX2 selective splicing [46].

## 7. LncRNAs Affect Decidualization and Energy Metabolism in PE

There have been numerous investigations suggesting that PE has an association with the dysplasia of decidualization [68]. Moreover, the poor decidualization can make a contribution to the decreased invasive potential of extravillous trophoblasts, dysplasia of the uterine spiral artery, and decreasing the blood flow at the maternal-fetal interface [69]. Placental ischemia results into the increased expression of toxic cytokines in the maternal peripheral blood, further impairing endothelial cells. Glycolysis is considered as quite pivotal for the development of endothelial cells, and decreased glycolysis was capable of contributing to the impaired decidualization [70]. The decreased level of HK2P1 and HK2 might be associated with the occurrence as well as the development of PE by means of the impairment of glycolysis and decidualization [71]. HK2P1 mediated the expression of HK2 by functioning as a competing endogenous RNA (ceRNA) [71]. These findings put forward innovative ideas regarding the etiology of PE, and the new regulating axis, which suggested HK2P1, HK2, and miR-6887-3p, might be put to use as the innovative indicators for PE [71]. Both PGK1 and PGK1P2 constitute a couple of ceRNAs related to miR-330-5p, in addition to being quite important in the mechanism of decidualization by means of the mediation of the angiogenesis and glycolysis [72]. Decreased expressions of PGK1 as well as PGK1P2 in the decidual tissues might give rise to the impaired decidualization and the occurrence of PE [72]. At present, increasing numbers of investigations have suggested that the occurrence of PE is associated with the abnormal energy metabolism. The expression of lncZBTB39-1:2 in placenta has the potential of decreasing the trophoblast activity by means of impacting the energy regulation, which may promote the progress of PE [73].

## 8. Conclusion

**Figure 1:**
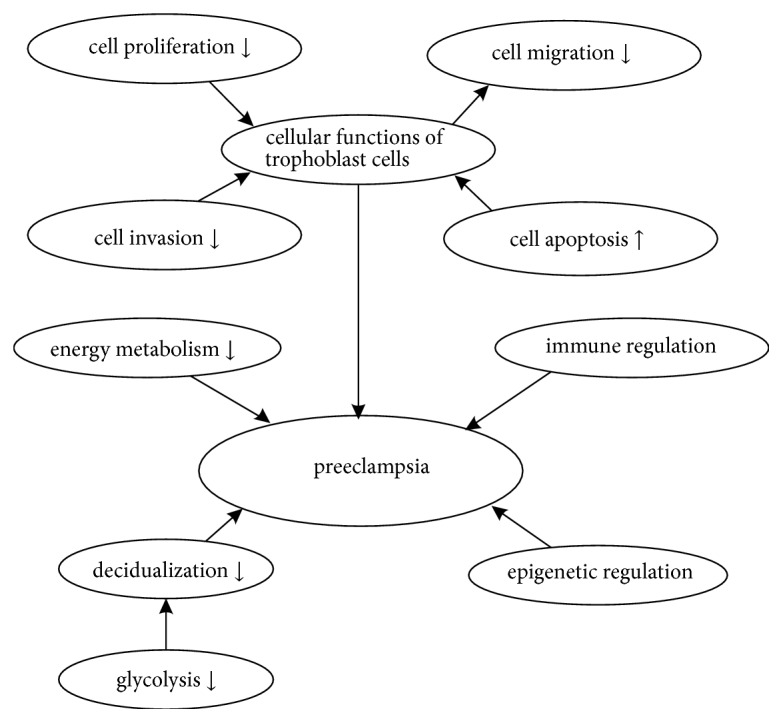
The biological functions of lncRNAs in PE. LncRNAs can exert an impact on both the occurrence and development of PE through the changes in the biological functions of trophoblasts (including cell proliferation, migration, invasion, and apoptosis), immune regulation, epigenetic regulation, decidualization, and energy metabolism, and impaired glycolysis may lead to the decreased decidualization.

In the current review, we have discussed about the latest research progress of lncRNAs and PE. According to the retrieved data, we concluded that lncRNAs can exert an impact on both the occurrence and development of PE through the changes in the biological functions of trophoblasts, immune regulation, epigenetic regulation, decidualization, and energy metabolism (Figure 1). Even though the functions of many lncRNAs are not clear, the mechanisms of lncRNAs in PE will help us to better understand the pathogenesis of PE and help us to find targets for predicting and diagnosing PE in the future.

## Figures and Tables

**Table 1:** Increased lncRNAs related to cellular functions of trophoblast cells in PE.

LncRNA name	Functions related to PE	Regulating gene/signal pathway	Reference
RPAIN	Inhibits invasion and promotes cell apoptosis	C1q	[40]
uc.187	Inhibits cell proliferation, invasion and increases cell apoptosis	unknown	[41]
CCAT1	Inhibits cell proliferation	CDK4	[42]
PRNCR1	Reduces cell viability	MAPK signaling pathway	[43]
DLX6-AS1	Decreases cell proliferation, migration and invasion	miR-376c/GADD45A axis	[44]
SPRY4-IT1	Decreases cell migration and proliferation, increases cell apoptosis	unknown	[45]
STOX2-IT3	Decreases cell invasion	STOX2	[46]
HELLP	Reduces cell differentiation, proliferation and invasion	YBX1, PCBP1, PCBP2, RPS6 and RPL7	[47, 48]
uc003fir	Reduces cell proliferation and migration	HIF1-*α*–lncRNA uc003fir–CCL5	[49]
CEACAMP8	unknown	unknown	[32]
LOC391533	unknown	unknown	[32]
LOC284100	unknown	unknown	[32]

MAPK, Mitogen-activated protein kinase.

**Table 2:** Decreased lncRNAs related to cellular functions of trophoblast cells in PE.

LncRNA name	Functions related to PE	Regulating gene/signal pathway	Reference
MALAT-1	Increases proliferation, decreases apoptosis	unknown	[50]
ATB	Increases migration, proliferation, tube-formation of HTR-8/SVneo cells	unknown	[51]
SNHG5	Promotes cell proliferation, invasion and migration	miR‐26a‐5p/N‐cadherin axis	[52]
MEG3	Reduces apoptosis and promotes migration	unknown	[53]
TUG1	Increases proliferation, migration, and invasion and reduces apoptosis	Ezh2, RND3	[54]
RP11-465L10.10	Increases cell migration and motility	MMP9	[30]
HOXA11-AS	Increases cell proliferation and migration	Ezh2, Lsd1, RND3 miR-15b-5p/HOXA7 axis	[55]
H19	Increases cell proliferation	miR-675/NOMO1 Nodal signaling	[56]
EGFR-AS1	Increases cell proliferation	JAK/STAT signaling pathway	[57]
linc00473	Increases cell proliferation and reduces cell apoptosis	TFPI2, LSD1	[58]
PVT1	Increases cell proliferation and reduces cell apoptosis	ANGPTL4, EZH2	[59]
MVIH	Promotes cell growth, migration, invasion and angiogenesis	Jun-B	[60]

SNHG5, small nucleolar RNA host gene 5; MEG3, maternally expressed gene 3; NOMO1, Nodal Modulator 1; EGFR-AS1, EGFR Antisense RNA1; TFPI2, tissue factor pathway inhibitor 2; LSD1, lysine-specific demethylase 1; ANGPTL4, angiopoietin-like 4.
